# Pure Large Cell Neuroendocrine Carcinoma of Ovary: A Rare Clinical Entity and Review of Literature

**DOI:** 10.1155/2012/120727

**Published:** 2012-12-06

**Authors:** P. N. Shakuntala, K. Uma Devi, K. Shobha, U. D. Bafna, M. Geetashree

**Affiliations:** ^1^Department of Gynaecologic Oncology, Kidwai Memorial Institute of Oncology, Dr. M. H. Mari Gowda Road, Bengaluru 560029, India; ^2^Department of Pathology, Kidwai Memorial Institute of Oncology, Dr. M. H. Mari Gowda Road, Bengaluru 560029, India

## Abstract

Large cell neuroendocrine carcinoma (LCNEC) of the ovary is a rare tumor and is now included in the World Health Organization tumor classification. Its prognosis is generally very poor even when the diagnosis is made at an early stage. We report a case of pure large cell neuroendocrine tumour of ovary, appearing 9 months following laparoscopic type I hysterectomy, bilateral pelvic lymph node dissection with ovarian preservation of anatomically normal looking ovaries performed for a cervical biopsy diagnosis of cervical intraepithelial neoplasia grade III with foci of invasion. The rarity lies in the rapid onset (9 months) of a large tumor following conservation of an anatomically normal ovaries. Surgical debulking and five cycles of chemotherapy (Etoposide and Cisplatin) were administered to the woman. She is on followup with no clinical or radiological evidence of disease recurrence for 6 months.

## 1. Introduction 

Ovarian pure large cell neuroendocrine (LCNEC) carcinoma is a rare tumour. Only 6 cases have been reported and all of them have been unilateral tumors. We report 7th case of pure LCNEC which was bilateral. They are rapid growing tumours with generally poor prognosis. Literature is replete with different modalities of adjuvant therapy. We have discussed the clinical presentation, histopathology, surgical debulking, and chemotherapy options and have also reviewed the literature.

## 2. Case Report

 A 40-year-old perimenopausal lady presented to us with a histopathological diagnosis of cervical intraepithelial neoplasia grade III with foci of invasion. A laparoscopic type I hysterectomy and bilateral pelvic lymphadenectomy along with conservation of normal looking ovaries was performed. She was clinically and radiologically free of disease on followup for 6 months. In the ninth month she presented with history of acute distension and pain abdomen, loss of weight and appetite, fever with chills, and itching all over the body. Her general condition was good. On abdominal examination a firm, irregular mass was felt occupying the suprapubic region and extending towards left iliac fossa, and left lumbar region, measuring 20 × 28 × 22 cms with restricted mobility and minimal ascites. On per vaginal and rectal examination, vault was healthy, and a firm, irregular abdominopelvic mass, measuring 25 × 28 × 22 cms, splaying the rectovaginal septum, with restricted mobility, rectal mucosa was free but thinned out. Bilateral parametria were supple. Both the pelvic side walls were free of tumor. Fine needle aspiration cytology of the mass revealed poorly differentiated malignant neoplasm. 

Complete haemogram, biochemistry, and chest X-ray were within normal limits. Ca-125 value was 280.80 IU/ml(high) and CEA was 7.66 ng/mL(high). Ultrasonography of abdomen and pelvis showed normal liver, gall bladder, and spleen except for left kidney with evidence of grade I hydroureteronephrosis secondary to compression by the mass. Uterus was not seen-post operative status. A large lobulated heterogenous mass lesion with few areas of necrosis in it, situated posterior to and indenting the base of the urinary bladder, measuring 9.5 × 12 × 20 cms extending laterally up to the left iliac fossa with minimal ascites was reported.

**Figure 1:**
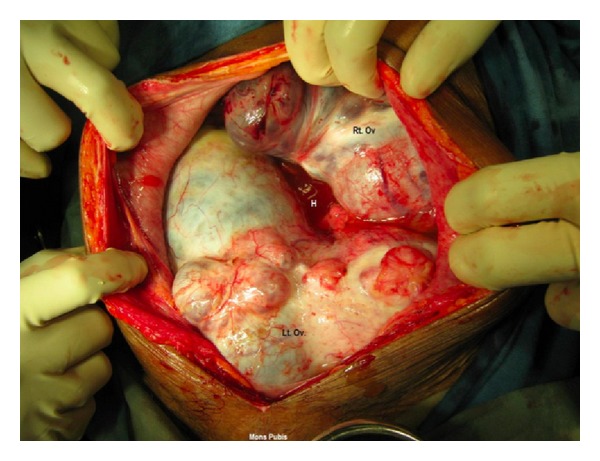
Rt.Ov—right ovarain tumour, Lt.Ov—left ovarian tumour adherent to bladder, lateral pelvic wall, rectum and vault and burrowing into the pouch of douglas. H: haemmorhagic ascites.

During surgery minimal haemorrhagic ascites was seen in the peritoneal cavity. Bilateral solid ovarian tumours with breech and deposits on the capsule were seen. The right ovarian tumour measuring 6 × 7 × 6 cms, adherent to the vaginal vault and pouch of douglas, and left ovarian tumour measuring 20 × 15 × 14 cms., solid and burrowing into the pouch of douglas adherent to rectum, vault, bladder base, and left lateral pelvic wall was released from the vital structures by sharp and fine dissection after tracing the ureters bilaterally, through a retroperitoneal approach Figure 1. On table frozen section of the ovarian mass revealed poorly differentiated carcinoma of ovary and metastatic poorly differentiated neoplasm of omentum. Then, followed by para-aortic lymph node dissection, total omentectomy, and removal of tumor deposits measuring 4 × 3 × 1 cms, over the sigmoid colon to achieve an optimal debulking. Subdiaphragmatic area, liver, gall bladder, stomach, spleen, rest of the intestines, and appendix appeared normal.

### 2.1. Pathology

Right ovarian tumour measuring 11 × 8 × 5 cms, capsule intact, surface shows nodularity, cut section shows solid, cystic, and firm. Left ovarian mass 11 × 9 × 7 cms, external surface was irregular and nodular, capsular breach was noted. Cut section shows haemorrhagic, solid and cystic areas. Omentum measured 28 × 6 × 3 cms Figure 1.

### 2.2. Microscopy

**Figure 2:**
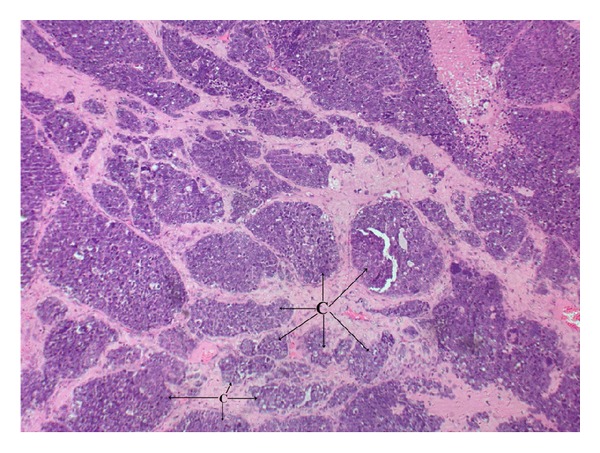
Neuroendocrine carcinoma shows clusters (C) of medium to large cells with moderate amount of cytoplasm and round to oval nuclei with even chromatin and occasionally prominent nucleoli (10x).

 Bilateral ovaries show poorly differentiated malignant tumour-carcinoma, with wide areas of necrosis. Mitotic rate 15–20/high power field, capsular breech noted in the left ovary. Omentum showed tumour deposits, and 3 paraaortic nodes were reactive. Bowel and bladder deposits showed tumour cells, Figure 2.

### 2.3. Immunohistochemistry

**Figure 3:**
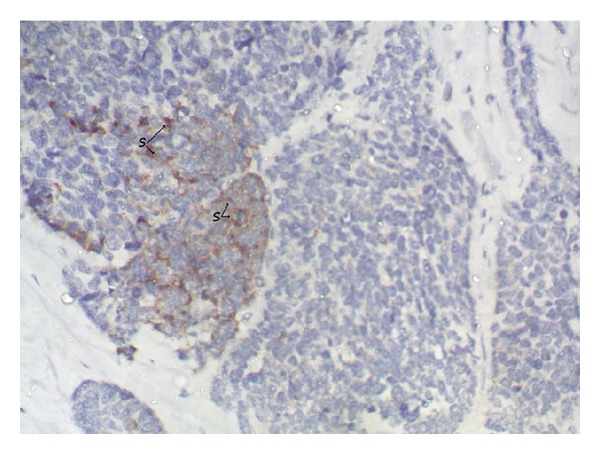
Cluster of neuroendocrine cells showing synaptophysin positivity(S) (40x).

CK, EMA were positive. CK 7, CK 20, Inhibin and Chromogranin were negative. Focal areas were positive for Synaptophysin, suggesting a final diagnosis of Large Cell neuroendocrine Carcinoma Ovary Figure 3.

Postoperative adjuvant chemotherapy consisting of Etoposide 100 mg/M2 from day 1 to day 5,and Cisplatin 100 mg/M2 in divided doses on day 1 and day 2 was administered 3rd weekly for 5 cycles. Patient tolerated chemotherapy, except for in-patient admission for neutropenic fever on day 7 during the 3rd and the 4th cycle. She was given symptomatic and supportive treatment. She is on followup for 6 months and has no clinical or ultrasonographic evidence of disease recurrence. 

## 3. Discussion 

The incidence of pure large cell neuroendocrine carcinoma (LCNEC) of ovary is very rare. Primary ovarian LCNEC is synonymous with undifferentiated carcinoma of non-small cell neuroendocrine type, according to the World Health Organization classification [7].

To date, only 40 cases of large cell neuroendocrine carcinoma (LCNEC) of the ovary are reported. 34 cases were associated with other histologic subtypes and 6 cases were purely LCNEC. They are generally associated with poor patient outcomes. The present case is the seventh case of pure form of ovarian large cell neuroendocrine carcinoma and the other rarity is, it is the first case arising bilaterally. This extremely rare clinical presentation of a huge mass arising from an anatomically normal ovary confirmed laparoscopically, 9 months back was reiterating the fact that this tumour was rapidly growing. Some authors have similar opinion [1–6].

 The age of patients was ranging between 27–81 years and a median age of 61 years. The commonest presentation was abdominal pain and distension in 50% (3/6) of cases, similarly the present case presented with abdominal distension. The size of the tumour ranged from 9 cms to 35 cms with an average of 16 cms. The tumor was bilateral in only the present case. Left side was more common in 50% of the cases (3/6). A range of therapeutic options have been used like surgery with chemotherapy, Table 1.

 As reported by Kim et al. [8], the pure forms of large cell neuroendocrine carcinoma displayed solid and cystic appearance on gross examination similar to the present case. Histopathologically, other epithelial tumour components were not identified except one case reported with squamous differentiation by Dundr et al. [3]. Immunohistochemically, these tumors were positive for neuroendocrine markers, such as CD56, chromogranin A, and synaptophysin. Synaptophysin was known to be more sensitive than chromogranin A [1]. The present case also showed positive immunoreactivity to synaptophysin, CK and EMA and negative immunoreactivity to CK7, CK 20, Inhibin, and chromogranin. On surgiopathlogic staging, she belonged to Stage III C.

Various combinations of chemotherapy were used, including cisplatin and cyclophosphamide followed by etoposide and cisplatin; or paclitaxel and carboplatin protocols Table 1. In the present case 5 cycles of etoposide 100 mg/M^2^ for 5 days along with cisplatin 100 mg/M^2^ divided doses on day 1 and day 2 every third weekly was administered. Survival data of the available patients: 5-year survival of the 33 LCNEC cases was 34.9% as discussed by Dundr et al. [3]. 

Three of the six patients have died of the disease immediately following surgery to 4 months after surgery. Present patient is on followup for last 6 months.

Out of the 6 cases of pure LCNEC, 2 cases had early stage disease and survived for a minimum of 10 months. In 3 cases there is no stage of disease mentioned but have had short survival of 4 months. One case had stage 4 disease and died after 2 months. Present case is a stage III disease who has survived for 6 months without evidence of recurrence, probably the longest Table 1.

Due to paucity of literature and heterogeneity in presentation and response to surgery, chemotherapy, and radiation it is oblivious to predict the efficacy of surgical debulking, chemotherapy and radiation protocols, hence there is a need for prospective clinical studies for most optimal modalities of treatment. May be a combination of optimal debulking, adjuvant chemotherapy (ifosamide, mesna, and cisplatin) with close followup of patients and use of radiation when appropriate may improve the progression free and survival rates of this rare tumour.

## Figures and Tables

**Table 1:** Clinicopathologic, treatment modality, and followup of women with pure large cell neuroendocrine tumour of ovary.

S. no.	Authors	Age/present.	Associated component	Site/laterality	Stage	Treatment modality	Followup
(1)	Behnam et al. [1]	27/pelvic mass	None	11 cm, left	Ic	LSO/omentectomy/ chemotherapy	NED 10 m
(2)	Lindboe [2]	64/abdominal discomport	None	14 cm, right	Ia	TAH/BSO/omentectomy/ chemotherapy	NED 9 m
(3)	Dundr et al. [3]	73/NA	None	9 cm, left	N/A	N/A	N/A
(4)	Aslam et al. [4]	76/abdominal pain	None	35, left	N/A	TAH/BSO/OMT	Died soon
(5)	Tsuji et al. [5]	46/abdominal distension	None (focal squamous differentiation)	12, right	N/A	TAH/BSO/OMT	Died in 4 m
(6)	Japan Oshita et al. (4 cases) [6]	42–81/N/A	Mixed epithelial carcinoma-3 cases, 1-none	N/A	Ic IIc IIIC IV	N/A, chemotherapy—paclitaxel and carboplatin	Died in 2 m Rest with or without recc-32–64 mo
(7)	Present case	40/abdominal dist., fever, itching	none	Bilateral ovarian 7 cm, Right 15 cm, Left	IIIc	BSO/TD/TO/ /PALND/ Bladder and sigmoid colon deposit excision + 5 cycles of etoposide (mesna) and cisplatin	NED-6 m

AWD: alive with disease; TAH: total hysterectomy, RSO: right salpingo-oophorectomy; BSO: bilateral salpingo-oophorectomy; LSO: left salpingo-oophorectomy; N/A: no information available; NED: no evidence of disease; NOS: not otherwise specified; DOD: dead of disease; m: months; y: years.
